# Enhanced Light-Induced Transverse Thermoelectric Effect in Tilted BiCuSeO Film via the Ultra-thin AuNPs Layer

**DOI:** 10.1186/s11671-019-3190-9

**Published:** 2019-12-05

**Authors:** Weiyuan Yu, Guoying Yan, Yuli Xue, Yuejiao Zhang, Jianglong Wang, Guangsheng Fu, Shufang Wang

**Affiliations:** Hebei Key Lab of Optic-Electronic Information and Materials, College of Physics Science and Technology, Hebei University, Baoding, 071002 China

**Keywords:** Light-induced transverse thermoelectric (LITT) effect, *c*-axis tilted BiCuSeO film, Ultra-thin AuNPs layer

## Abstract

Significant enhancement of light-induced transverse thermoelectric (LITT) effect in tilted BiCuSeO film has been achieved via introduction of an ultra-thin layer of gold nanoparticles (AuNPs) with the thickness of a few nanometers. In both cases of pulsed and continuous light irradiation, about two times increment in the LITT voltage sensitivity is observed for the BiCuSeO film coated with 4-nm-thick AuNPs layer. This can be ascribed to the increased photo-thermal conversion efficiency in the LITT effect owing to the efficient usage of the incident light of AuNPs layer. Thicker AuNPs layer will suppress the voltage sensitivity increment due to the electrical connectivity effect. This work provides an effective strategy for optimizing the performance of thermal-type optical detectors based on the LITT effect.

## Introduction

**Fig. 1:**
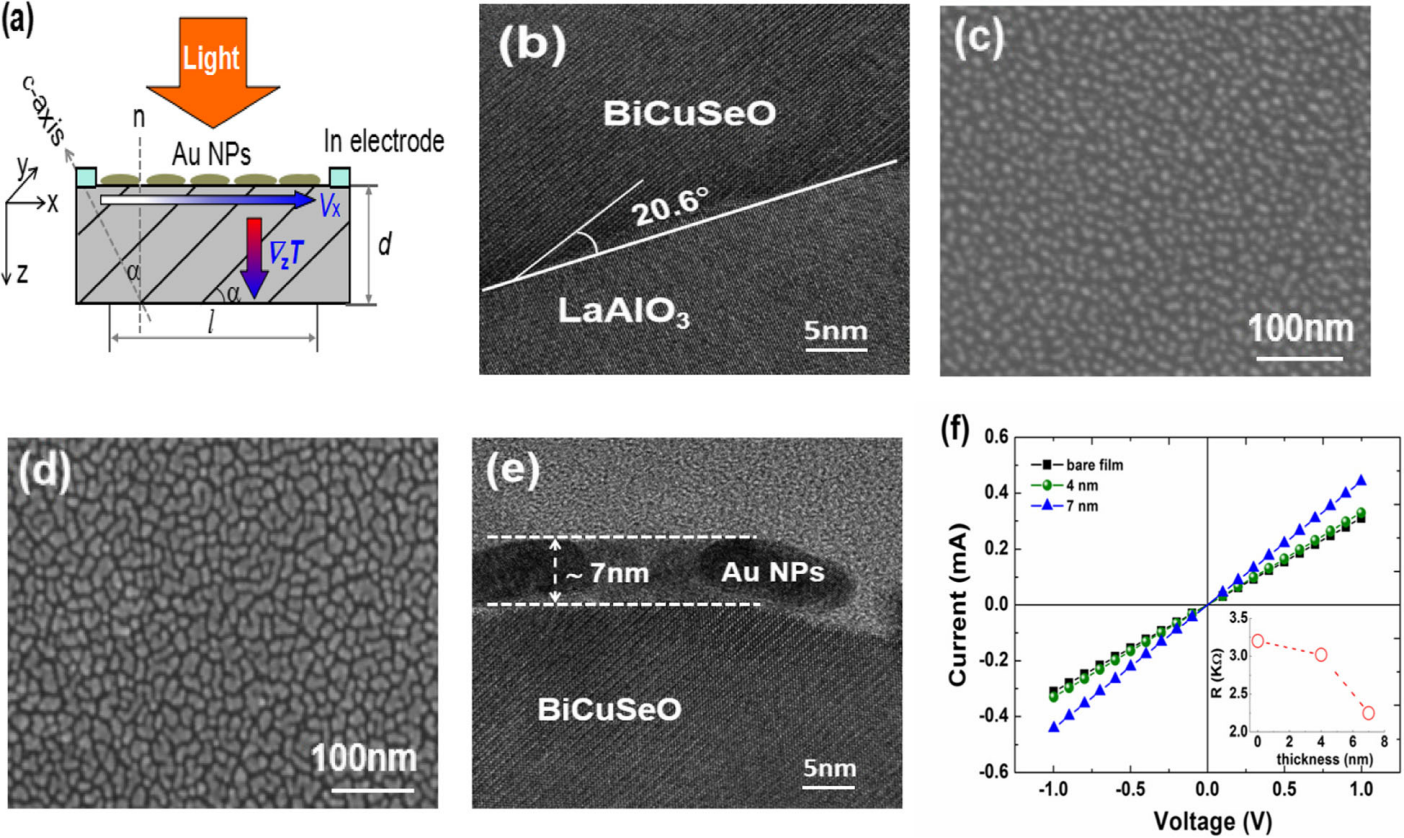
**a** Schematic illustration of the LITT effect in a *c*-axis tilted film coated with AuNP layer. **b** HRTEM image of a BiCuSeO film grown on 20° miscut LaAlO_3_ (001) substrate. **c**–**d** SEM images of the AuNP layer with thickness of 4 and 7 nm, respectively. **e** HRTEM image of the sample of AuNP (7 nm)/BiCuSeO. **f** *I*–*V* curves between two indium electrodes on different samples. The inset is the variation of resistance of AuNPs/BiCuSeO samples with the AuNP layer thickness

Light-induced transverse thermoelectric (LITT) effect is a special thermoelectric phenomenon in which the electric and heat fluxes in the material are perpendicular to each other. This effect originates from the anisotropy of Seebeck coefficient and can only be detected in tilted structures [1, 2]. As shown in Fig. 1a, when the surface of a *c*-axis tilted film is illuminated by light, a temperature difference Δ*T*_z_ between the film surface and the bottom is established along the *z*-axis because of the absorption of incident light, which will result in a thermal voltage signal *V*_x_ along the *x*-axis direction. The induced voltage *V*_x_ can be expressed as:

1 $$ {V}_x=\frac{l}{2d}\sin \left(2\alpha \right)\cdot \varDelta S\cdot \varDelta {T}_z $$

where *l*, *d*, and *α* are the light spot diameter on the film, the film thickness, and the tilted angle of the *c-*axis with respect to the film surface normal, respectively. Δ*S* = *S*_ab_ − *S*_c_ is the difference of Seebeck coefficient in *ab*-plane and along the *c*-axis direction of the film [2].

In the past few years, LITT effect has attracted great attention due to the potential applications in the self-powered uncooled optical detectors. Extensive studies have been carried out on tilted films of YBa_2_Cu_3_O_7-δ_, La_1-x_Ca_x_MnO_3_, Ca_x_CoO_2_, Bi_2_Sr_2_Co_2_O_y_, La_0.9_Sr_0.1_NiO_3_, SrTi_1−x_Nb_x_O_3_, etc. [3–14]. However, the voltage sensitivity *R*_s_, which is defined as the ratio of the output voltage amplitude *V*_p_ to the incident light energy *E* irradiated on the film, obtained from these films is not yet sufficient for practical applications in optical detectors. Recently, to improve *R*_s_, a layer of gold black or carbon nanotubes with the thickness of a few micrometers (μm) was coated on the film surface by Takahashi et al. and Wang et al. [15–18]. The gold black or carbon nanotube layer can act as the light absorption layer, which is expected to improve the photo-thermal conversion efficiency of the LITT effect and increase the value of Δ*T*_z_. This strategy was proved to be very effective for continuous light irradiation. While for pulsed light irradiation, the introduction of micrometer-thick light absorption layer resulted in a significant deterioration in *R*_s_, reducing to only about 0.5% of the original value. Although the micrometer-thick light absorption layer increases the utilization of the incident light, it greatly suppresses the input thermal energy of the pulsed light irradiation because of the excessively prolonged thermal relaxation time in the whole system, which eventually leads to a decreased Δ*T*_z_ [15]. The ultra-thin gold nanoparticles (AuNPs) layer plays a quite significant role in material science due to its unique chemical and physical properties, which has been widely used in many fields such as photonics, solar harvesting, biological sensing, surface-enhanced Raman scattering, and molecular spectroscopy applications [19–21]. In this paper, we explored the usage of ultra-thin AuNPs layer, with a thickness of 4–7 nm, as light absorption layer to enhance the voltage sensitivity *R*_s_ of LITT effect in the tilted film of BiCuSeO. This compound is a new promising thermoelectric material with an anisotropic layered structure [22–25], which makes it a good candidate material for the study of the LITT effect [26, 27]. As the thermal relaxation process in the ultra-thin AuNPs layer is very fast and can be ignored, the thermal relaxation process in the present AuNPs/BiCuSeO system is still governed by BiCuSeO film. In both cases of continuous and pulsed light irradiation, about two times increment in *R*_s_ has been achieved by sputtering a 4-nm-thick AuNPs layer on the BiCuSeO film. When the thickness of AuNPs layer increases to about 7 nm, the contribution of the AuNPs layer to the resistivity of the whole structure (Au/BiCuSeO) cannot be ignored anymore due to its good electrical conductivity, which will suppress the increment of *R*_s_.

## Methods

### Preparation of BiCuSeO Film and AuNPs Layer

In this work, *c-*axis tilted BiCuSeO films with thickness of about 150 nm were fabricated by using a 308-nm pulsed laser ablation of the BiCuSeO ceramic target under an atmosphere of high purity argon. The tilted angle of the film was regulated by the miscut angle of the substrate. Here, 20° miscut (001) LaAlO_3_ single crystal substrates were used. Details of film fabrication and structural characterization can be found in our previous papers [25–27]. AuNPs layer, with a thickness of 4 and 7 nm respectively, was coated on the tilted BiCuSeO film by sputtering technique. During the sputtering process, the Ar gas pressure in the chamber was set at 0.1 Pa, the substrate temperature was maintained at 300 K, and the sputtering current was 6 mA.

### Characterization

SEM and HRTEM were used to illustrate surface and cross-section images of the AuNPs layer. To estimate the light absorption and photothermal conversion characteristics of the BiCuSeO film as well as the ultra-thin AuNPs layer, the light absorption spectra of bare BiCuSeO, AuNPs layer, and AuNPs/BiCuSeO were measured by using a Hitachi U-4100 spectrophometer, respectively.

### Thermoelectric Performance

We performed electrical resistivity *ρ* and Seebeck coefficient *S* measurements on the BiCuSeO film with carrier density of about 6.6 × 10^−19^ cm^−3^, as shown in Additional file 1: Figure S1. At room temperature, the *ab*-plane electrical resistivity and Seebeck coefficient of the BiCuSeO film were about 11.5 mΩ cm and 204 μV/K, resulting in a power factor of about 0.36 mW/mK^2^. The out-of-plane thermal conductivity of this film sample was measured by the Linseis thin film Laser flash Analyser (TF-LFA), and it was about 0.24 W/mK at room temperature.

### LITT Effect Measurement

For the measurement of LITT effect, two indium electrodes separated about 8 mm were deposited on the film surface along the *x*-axis direction, as shown in Fig. 1a. A 308-nm pulsed laser with an energy density of 0.2 mJ/mm^2^ and a Xenon lamp with a power density of 350 mW/cm^2^ were used as the light sources. To avoid Dember effect, the light spot (3 mm × 5 mm) on the film was located on the center position between two electrodes. The LITT voltage signals were recorded by a digital oscilloscope terminated into 1 MΩ (Agilent DSO9254A) and a 2700 Keithley source meter for pulsed and continuous light irradiations, respectively.

## Results and Discussion

Figure 1b presents the HRTEM image of BiCuSeO film grown on a 20° miscut LaAlO_3_ (001) substrate. It can be clearly seen that the film grows along the *c*-axis and its *c*-axis is tilted about 20° away from the film surface normal. Figure 1c and d display the SEM surface images of the 4- and 7-nm-thick AuNPs layer, respectively. The AuNPs form a continuous gold layer, in which the AuNPs are in contact with each other but are not totally fused. The average size of AuNPs is below 10 nm for the 4-nm-thick AuNP layer, and it grows bigger when the thickness of the film increases to 7 nm. XRD measurement of both AuNPs layers shows no obvious diffraction peak from Au, indicating amorphous feature of the AuNPs layer. Figure 1e presents the cross-section HRTEM image of AuNPs (7 nm)/BiCuSeO interface, indicating the good contact between the AuNPs and the BiCuSeO film surface. We believe that the very thin thickness of the AuNPs layer as well as the good AuNPs/BiCuSeO interface will be helpful for suppressing the thermal relaxation time of the input heat energy in the LITT effect, which will be very important for the pulsed light irradiation. Figure 1f demonstrates the current-voltage (*I*-*V*) curves between two electrodes on the tilted BiCuSeO film, in which linear conductive behavior confirms perfect Ohmic contacts between the electrode and the film. The inset of Fig. 1f shows the resistance of AuNPs/BiCuSeO. It decreases from 3.2 KΩ for bare BiCuSeO to 3.02 KΩ for 4-nm-thick AuNPs/BiCuSeO and 2.25 KΩ for 7-nm-thick AuNPs/BiCuSeO. The reduction in resistance is suggested to originate from the contribution of the AuNPs layer. As the thickness of AuNPs layer increases, it becomes more electrically conductive, resulting in a decreased resistance of the whole AuNPs/BiCuSeO structure.

**Fig. 2:**
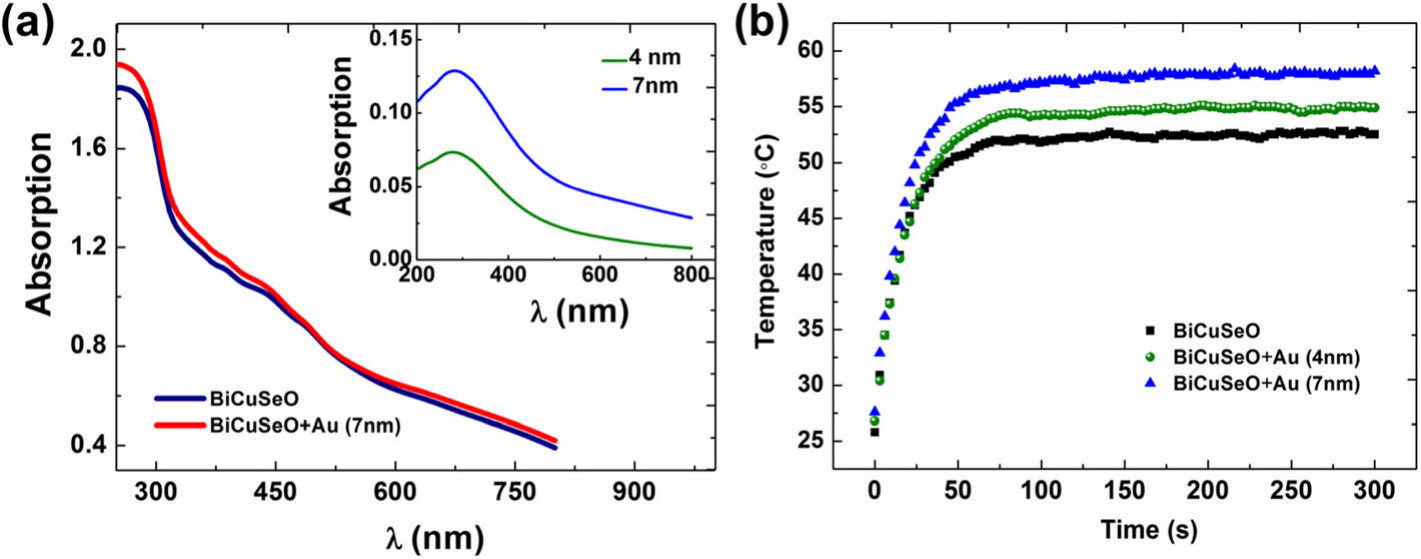
**a** Light absorption spectra of bare BiCuSeO and AuNPs (7 nm)/BiCuSeO samples. The inset is the light absorption spectra of Au layer with a thickness of 4 nm and 7 nm. **b** Heating curves of bare BiCuSeO and AuNPs/BiCuSeO samples under the Xenon lamp illumination

Figure 2a displays the light absorption spectrum of BiCuSeO film before and after coating the AuNPs layer. The introduction of a few nanometer-thick AuNP layer only leads to a slight increment in the light absorption because of the high transmittance of the ultra-thin AuNPs layer. To give more information, the light absorption spectrum of the 4- and 7-nm-thick AuNPs layers are also presented in the inset of Fig. 2a. The peak at about 280 nm (~ 4.4 eV) originates from the inter-band transition, which corresponds to the L gap of gold [28]. It should be mentioned here that the AuNPs in the ultra-thin layer are not separated but are in contact with each other. Therefore, we did not observe the plasmon resonance peak of AuNPs around 550 nm as well as the spectral shift between the peaks of the two layers when increasing the amount of gold.

To estimate the effect of such ultra-thin AuNPs layer on the photo-thermal conversion efficiency of BiCuSeO films, we measured the heating curves of bare BiCuSeO as well as AuNPs/BiCuSO samples upon the Xenon lamp irradiation, which are shown in Fig. 2b. It can be clearly seen that the ultra-thin AuNPs layer is very effective for improving the photo-thermal conversion efficiency of the BiCuSeO film in spite of the slight increment in the light absorption. The steady-state temperature of the sample surface increases from 52 °C for bare BiCuSeO to 55 °C for 4-nm-thick AuNP layer/BiCuSeO and 58 °C for 7-nm-thick AuNP layer/BiCuSeO. This is probably due to the fact that the heat capacity *C*_p_ of AuNPs (27 Jmol^−1^ K^−1^) is much smaller than that of BiCuSeO (99.5 Jmol^−1^ K^−1^), leading to a higher temperature rise when absorbing a similar amount of light energy [29, 30]. In addition, the introduction of the amorphous AuNP layer may reduce the reflectance loss of light at the smooth BiCuSeO film surface. All these effects sum up to increase the vertical temperature gradient established in the BiCuSeO film.

**Fig. 3:**
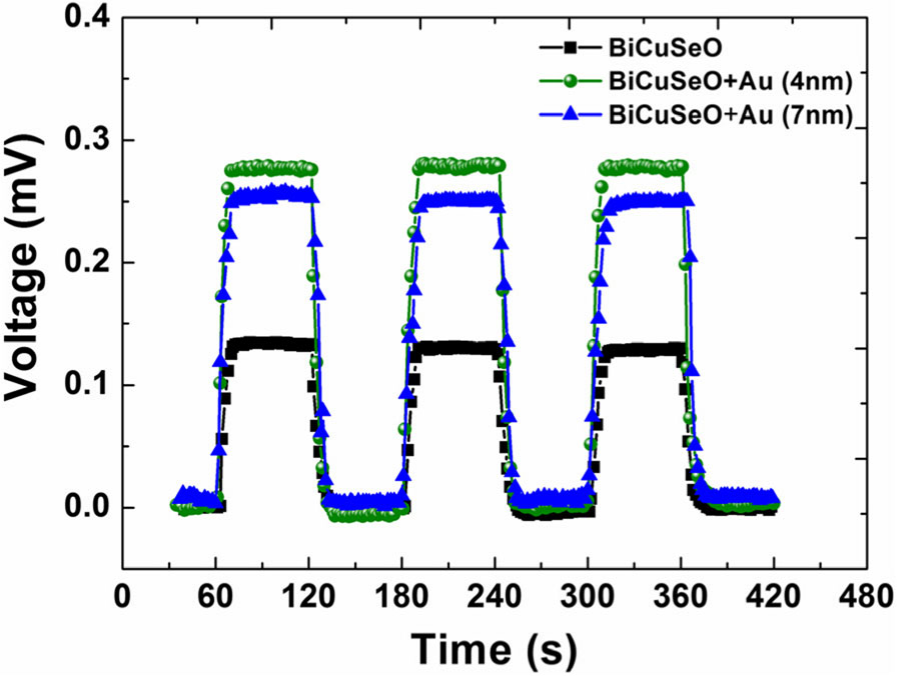
Voltage responses of bare BiCuSeO and AuNPs/BiCuSeO samples upon a Xenon illumination

Figure 3 illustrates the voltage responses of the tilted BiCuSeO films with and without coating the ultra-thin AuNPs layer upon the illumination of a Xenon lamp. As the light is turned on, open-circuit voltage signals are detected in all samples. Moreover, the magnitude of the light-induced voltage signal, *V*_p_, increases significantly after introducing the ultra-thin AuNPs layer. For example, for the BiCuSeO film with the 4-nm-thick AuNPs layer, the value of *V*_p_ is 0.27 mV, which is about two times larger than that of the bare film (0.13 mV). This result reveals that the ultra-thin AuNPs layer in a few nanometers thick can greatly enhance the voltage sensitivity *R*_s_ of the LITT effect under the continuous light radiation.

**Fig. 4:**
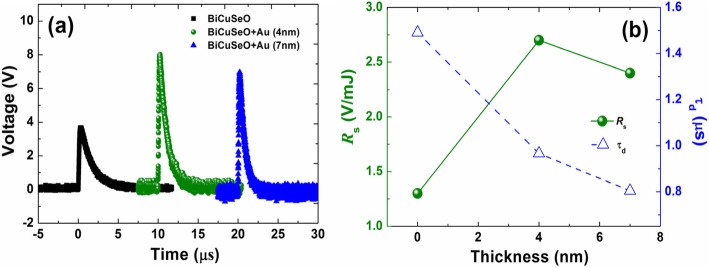
**a** Voltage responses of bare BiCuSeO and AuNPs/BiCuSeO samples upon a 308-nm pulsed laser illumination. **b** Voltage sensitivity *R*_s_ and decay time *τ*_d_ of these voltages

To check whether the ultra-thin AuNPs layer is also effective in the case of pulsed light radiation, we performed the LITT measurements by using a 308-nm pulsed laser as the light source. Figure 4 a is the voltage responses of the film samples upon the pulsed light radiation. The pulsed light-induced voltage signal in the tilted BiCuSeO film is also greatly enhanced after coating the ultra-thin AuNPs layer. The value of *V*_p_ increases from 3.8 V for bare BiCuSeO to 8.1 V for the film coated with the 4-nm-thick AuNP layer, resulting in an improvement of *R*_s_ from 1.3 to 2.7 V/mJ, as shown in Fig. 4b. In addition to *R*_s_, decay time *τ*_d_, always obtained by fitting the attenuation portion of the induced voltage signal, is another important parameter to evaluate the characteristics of LITT effect for pulsed laser source. It is clear that *τ*_d_ in Fig. 4b monotonously decreases from 1.5 μs for bare BiCuSeO to 0.8 μs for 7-nm-thick AuNPs/BiCuSeO. The reduction in *τ*_d_ is different from the report in, and it may be caused by the ultra-thin structure as well as the electrical connectivity effect of the AuNPs layer.

**Fig. 5:**
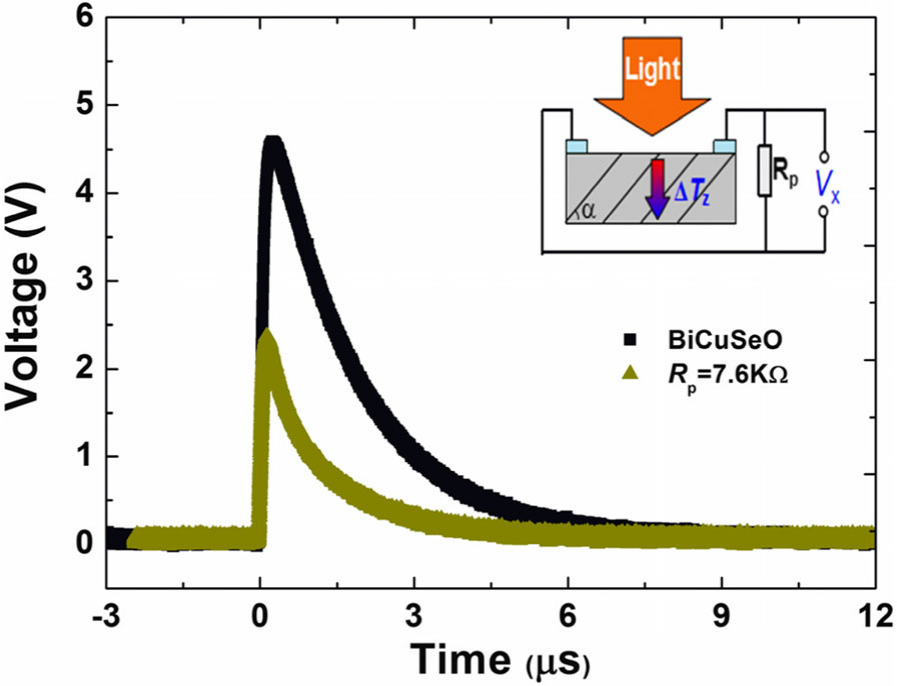
Voltage responses of bare BiCuSeO upon a 308 nm pulsed laser illumination before and after connecting a 7.6 KΩ resistor in parallel

It should be noted here that in both cases of continuous and pulsed light irradiation, the value of *R*_s_ shows a downward trend when the thickness of AuNPs layer increases to 7 nm though it is still higher than the original value obtained from the bare film. This behavior may be due to the parallel effect of AuNPs layer. It is known that connecting a parallel resistor with small resistance in the measurement circuit will lead to a reduced *V*_p_ and a faster response time [8, 10, 30]. In this work, the ultra-thin AuNPs layer can be regarded as a resistor connected in parallel with the BiCuSeO film. As the thickness of AuNPs layer increases from 4 to 7 nm, its resistance decreases from 54 to 7.6 KΩ. As shown in Fig. 5, connecting a 7.6 KΩ resistor in parallel with the BiCuSeO film indeed results in the reduction in both amplitude and decay time *τ*_d_ of the output voltage signal. In order to verify the rationality of the explanation, we also performed the LITT measurement on a sample with 20-nm-thick AuNPs layer under the illumination of the 308 nm pulsed laser: here, the AuNPs layer is continuous and shows a smaller resistance in comparison with the 4 or 7 nm-thick film. As the thickness of AuNPs layer increases, the values of *V*_p_ as well as *τ*_d_ continue to drop (as seen in Additional file 1: Figure S2).

## Conclusions

In conclusion, an ultra-thin AuNPs light absorption layer with the thickness of a few nanometers was introduced to improve the voltage sensitivity of the LITT effect in the *c*-axis tilted BiCuSeO film. In both cases of continuous and pulsed light irradiation, the magnitude of the output voltage signal (*V*_p_) of the LITT effect increased more than two times after sputtering a 4-nm-thick AuNPs layer on the tilted BiCuSeO film. This can be ascribed to the enhanced photo-thermal conversion efficiency of the AuNPs/BiCuSeO structure. However, when the thickness of the AuNPs layer became thicker, the increased electrical connectivity effect of the AuNPs layer suppressed further improvement of *R*_s_. These results can provide some useful guidance for designing high-performance thermal-type optical detectors based on the LITT effect.

## Additional File

**Additional file 1: Figure S1.** The temperature dependence of *ab*-plane (a) resistivity ρ_ab_ and (b) Seebeck coefficient *S*_ab_ of untilted BiCuSeO film. **Figure S2.** (a) SEM surface image of a 20 nm-thick AuNPs layer and (b) Voltage response of AuNPs(20 nm)/BiCuSeO to the 308 nm pulsed light irradiation. For comparison, data of bare BiCuSeO is also provided.

## Data Availability

All data are fully available without restriction.

## Abbreviations

*τ*_d_: Decay time of the induced voltage
AuNPs: Gold nanoparticles
HRTEM: High-resolution transmission electron microscopy
LITT: Light-induced transverse thermoelectric
*R*_s_: Voltage sensitivity
SEM: Scanning electron microscope
*V*_p_: Magnitude of the induced voltage
