# Supplementary material for: Enhanced Light-Induced Transverse Thermoelectric Effect in Tilted BiCuSeO Film via the Ultra-thin AuNPs Layer
Source: Nanoscale Res Lett. 2019 Dec 5;14:367. doi: 10.1186/s11671-019-3190-9 (PMC6895342; doi:10.1186/s11671-019-3190-9)
Supplement: Supplementary file 1 — Additional file 1: Figure S1. The temperature dependence of ab-plane (a) resistivity ρab and (b) Seebeck coefficient Sab of untilted BiCuSeO film. Figure S2. (a) SEM surface image of a 20 nm-thick AuNPs layer and (b) Voltage response of AuNPs(20 nm)/BiCuSeO to the 308 nm pulsed light irradiation. For comparison, data of bare BiCuSeO is also provided. [file 11671_2019_3190_MOESM1_ESM.docx]

Enhanced light-induced transverse thermoelectric effect in tilted BiCuSeO film via the ultra-thin AuNPs layer (SUPPLEMENTARY MATERIAL)

Weiyuan Yu, Guoying Yan*, Yuli Xue, Yuejiao Zhang, Jianglong Wang, Guangsheng Fu,

Shufang Wang*

*Hebei Key Lab of Optic-Electronic Information and Materials, College of Physics Science and Technology, Hebei University, Baoding 071002, China*

**Fig. S1.** The temperature dependence of *ab*-plane (a) resistivity ρ_ab_ and (b) Seebeck coefficient S_ab_ of BiCuSeO film.




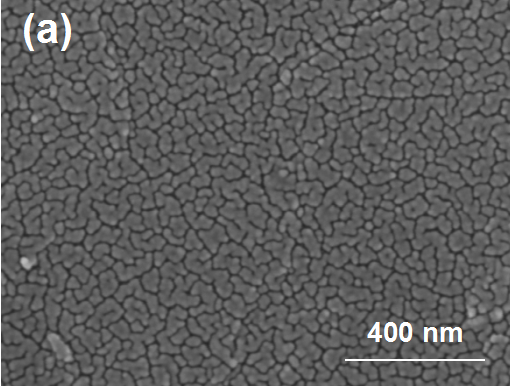



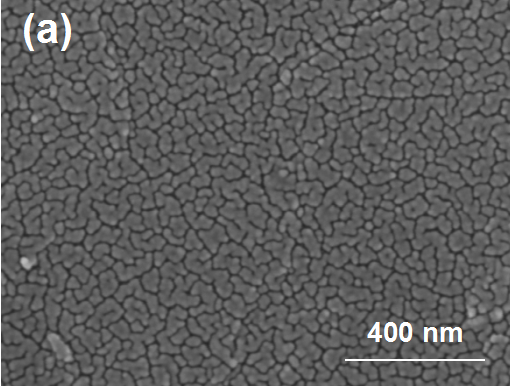


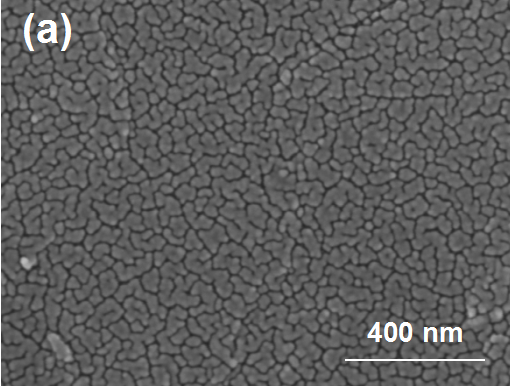


**Fig. S2.** (a) SEM surface image of a 20 nm-thick AuNPs layer and (b) Voltage response of AuNPs(20 nm)/BiCuSeO to the 308 nm pulsed light irradiation. For comparison, data of bare BiCuSeO is also provided.
